# Genipin—Simple but Significant Bioactive Iridoid for Therapeutical Application and Beyond: A Review

**DOI:** 10.3390/life15020159

**Published:** 2025-01-23

**Authors:** Danuta Sobolewska, Agnieszka Galanty, Karolina Grabowska, Justyna Makowska-Wąs, Irma Podolak, Dagmara Wróbel-Biedrawa

**Affiliations:** Department of Pharmacognosy, Medical College, Jagiellonian University, 30-688 Cracow, Poland; danuta.sobolewska@uj.edu.pl (D.S.); agnieszka.galanty@uj.edu.pl (A.G.); karolina1.grabowska@uj.edu.pl (K.G.); justyna.makowska-was@uj.edu.pl (J.M.-W.); irma.podolak@uj.edu.pl (I.P.)

**Keywords:** genipin, anti-inflammatory, anti-proliferative, blue pigment, cross-linking agent

## Abstract

Genipin is a non-glycosidic iridoid isolated mainly from the fruits of *Gardenia jasminoides* and *Genipa americana*. It is the active ingredient in extracts from these plants, responsible for their anti-inflammatory and hepatoprotective effects. In several in vitro tests, its anti-proliferative activity against tumour cell lines has been demonstrated, and due to its ability to specifically inhibit the UCP2 protein and inhibit STAT3 activation, a significant increase in the cytotoxicity of several anticancer drugs was observed in co-treatment with genipin. In recent years, the importance of genipin has increased due to the possibility of using this iridoid as a biocompatible and low cytotoxicity potent crosslinking agent in the manufacture of dressings, in tissue engineering, as a component of a drug carrier system and in the production of food packaging. Genipin is also a substrate in the production of a blue pigment used as a food additive and fabric pigment, and other applications. Due to documented cases of hepatotoxicity, genipin and the blue pigment derived from it are being investigated for effective and safe therapeutic and non-drug use. The current paper discusses selected aspects of chemistry, activity and use of this interesting compound.

## 1. Introduction

**Figure 1:**
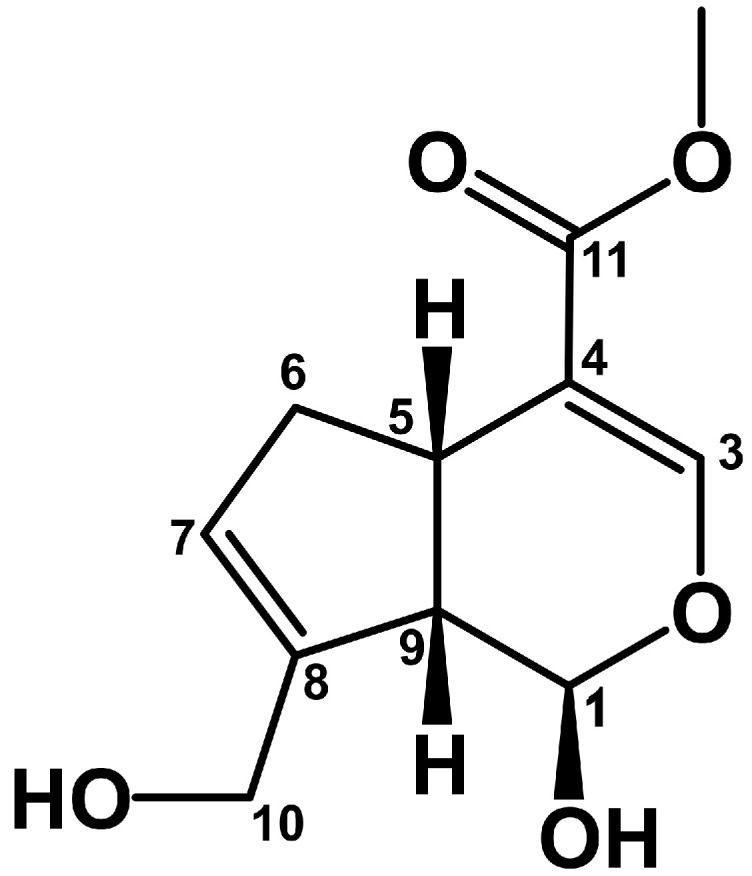
Structure of genipin.

Genipin, according to the IUPAC nomenclature: methyl (1R,4aS,7aS)-1-hydroxy-7-(hydroxymethyl)-1H,4aH,5H,7aH-cyclopenta[c]pyran-4-carboxylate (Figure 1), is a representative of the iridoids, a group of monoterpene compounds with a basic cyclopentane-α-pyran skeleton. These compounds are generally classified as secondary/specialised metabolites of plants, although the term ‘iridoids’ itself is derived from the generic name of the *Iridomyrmex* ants, in whose defence secretions these monoterpenes were originally identified [1].

Approximately 800 iridoids have been described to date [2]. They have been identified in 57 botanical families [3]. In some of them, such as Plantaginaceae, Bignoniaceae, and Oleaceae, these compounds are indicated as chemotaxonomic markers. Among insects, iridoid compounds have been detected in representatives of beetles, hymenoptera, and bugs [4]. Insect larvae have been shown to synthesise new compounds de novo; some can also sequester phytogenic precursors [5].

**Figure 2:**
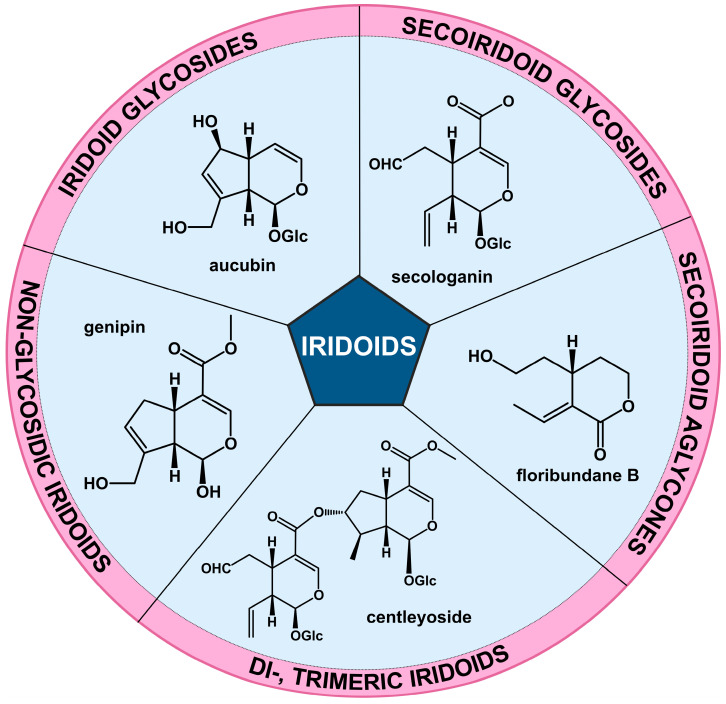
Classification of iridoids.

Although iridoids were first isolated in the 19th century, their structure was not actually elucidated until the mid-20th century [1]. On its basis, iridoids are classified into several sub-groups: non-glycosidic iridoids, iridoid glycosides, secoiridoids (both free and glycosidated) and oligomeric iridoids—di- and trimeric compounds (Figure 2) [3].

Genipin belongs to the non-glycosidic, ‘simple’ iridoids, with an unmodified basic 10-carbon skeleton. In nature, it occurs as a free compound or, more commonly, in a glycoside form (e.g., genipin 1-*O*-β-D-glucopyranoside—geniposide, genipin 1-*O*-β-D-gentiobioside) [6,7].

It was first isolated by Djerassi et al. in 1960 from the fruit of *Genipa americana* L. (Rubiaceae), a plant also known as Genipap, which occurs naturally in Central and South America [8,9]. Later it was also identified in approximately 10 plant species, among others, in the bark of *Apodytes dimidiata* E.Mey. ex Arn.; (Metteniusaceae), the bark of *Eucommia ulmoides* Oliv. (Eucommiaceae), fruit of *Gardenia jasminoides* J.Ellis (Rubiaceae), inflorescences of *Garrya elliptica* Douglas ex Lindl. (Garryaceae), fruit of *Rothmannia globosa* (Hochst.) Keay and fruit and bark of *R. witti* (Rubiaceae) [10,11,12,13,14]. In contrast, the presence of its 1-*O*-β-D-glucopyranoside (geniposide) has been reported in more than 40 plant species, mainly belonging to the Rubiaceae family [7]. The synthesis of genipin was first carried out in 1967 by Büchi et al. [15].

Genipin and its plant sources are of great research interest because of the broad spectrum of reported pharmacological activities. Most importantly, they are the subject of extensive studies owing to the possible use of genipin as a biocompatible, non-cytotoxic cross-linking agent, as well as its application as a substrate for the production of blue pigments for food and non-food purposes.

Genipin (C_11_H_14_O_5_; 226.23 g/M; CAS 6902-77.8) is a crystalline colourless substance with a melting point of 120–121 °C. Its solubility in water at 25 °C is 1% (*w*/*v*), while at 37 °C it is 2%. The compound is soluble in ethanol, DMSO, and dimethylformamide. Evaluation of the colour of aqueous genipin solutions at different pH values: 1.2, 5.0, 7.4, and 9.0 showed that they were colourless in this range, while at pH 13.6 they took on a brown colour and became viscous [16]. At high pH values, genipin ring opening occurs with the formation of an aldehyde form and polymerisation by aldol condensation with the formation of macromers composed of 7–88 mers, whose molecular weight can reach 20 kDa. Degradation of the compound was monitored in aqueous solutions at different pH values by HPLC [17]. The process was more accentuated at high pH values, and an increase in the stability of the compound was observed when the pH decreased from a value of 9 to 6; at pH 4 and 5, the stability of iridoid was comparable to that at pH 7. The authors confirmed the earlier thesis, explaining the degradation of genipin as a reversible opening of the dihydropyran ring by water followed by irreversible polymerisation of the intermediate.

Genipin, in the presence of oxygen, reacts with primary amino groups of, for example, amino acids, peptides, and proteins, with the formation of a blue product [18,19]. Running this reaction under anaerobic conditions in a nitrogen atmosphere first gave a yellow and then a brownish-red reaction mixture, which took on a blue colour in the presence of oxygen [20]. Blue genipin-derived pigments have been commonly used as natural food colourants in East Asia.

Though genipin has been obtained synthetically, isolation from plant sources is still a preferred mode of its acquisition [15]. The compound is most commonly extracted from the unripe fruits of *Genipa americana* and fruits of *Gardenia jasminoides.* The isolation of free genipin from plant material is a multi-step and often costly process. It has been extracted from unripe *G. americana* fruit with organic solvents, e.g., chloroform or methanol (genipin content in methanol extract 60.77 mg/g freeze-dry-weight), followed by a time-consuming, multi-step purification process [21,22,23]. Therefore, to date, the compound is most often obtained indirectly—by hydrolysis of geniposide, which is present in the plant at higher concentrations than the free aglycone [23,24].

In various studies β-glucosidases from fungi, e.g., *Aspergillus niger, Penicillium nigricans*, and *Trichodrema harzianum*, have been used to hydrolyse the glycosidic bond of geniposide [23]. Genipin was extracted from the dry fruits of *Gardenia jasminoides* with high efficiency (58.83 mg/g dry weight) by enzyme-assisted extraction and in situ product separation technique, using cellulase as a biocatalyst and ethyl acetate as an environmentally friendly extraction solvent [25]. In another study, genipin was obtained from unripe *Genipa americana* fruit using enzyme-assisted extraction in a liquid–liquid two-phase aqueous system [23]. The compound was isolated also from Genipap peel by mechanical/sonic enzyme-assisted extraction at low temperatures [22]. After the cold extraction step, genipin was separated from proteins and pectins using pectinesterase. A combination of ultrasound and/or microwave pretreatments followed by enzymatic hydrolysis and simultaneous extraction (EHSE) and ionic liquid-based enzyme-assisted extraction coupled with in situ hydrolysis (ILEIH) of geniposide was proposed for obtaining genipin from geniposide present in the bark of *Eucommia ulmoides* [26,27].

## 2. Materials and Methods

We performed an extensive search for relevant references indexed in the PubMed database up to November 2024, using the following keywords: genipin, iridoids, gardenia blue, blue pigment, anti-inflammatory, anti-proliferative, hepatoprotective, hepatotoxicity, cross-linking agent, bioavailability, colorimetric analyses. Additional searches, mainly for related articles, were performed in Google Scholar, Scopus, Web of Science, and other professional websites. We assessed 168 publications for relevance and included them in the current study. Plant names were verified in the Plants of the World Online database (https://powo.science.kew.org/, accessed on 23 November 2024) [9].

## 3. Pharmacological Activity of Genipin

**Figure 3:**
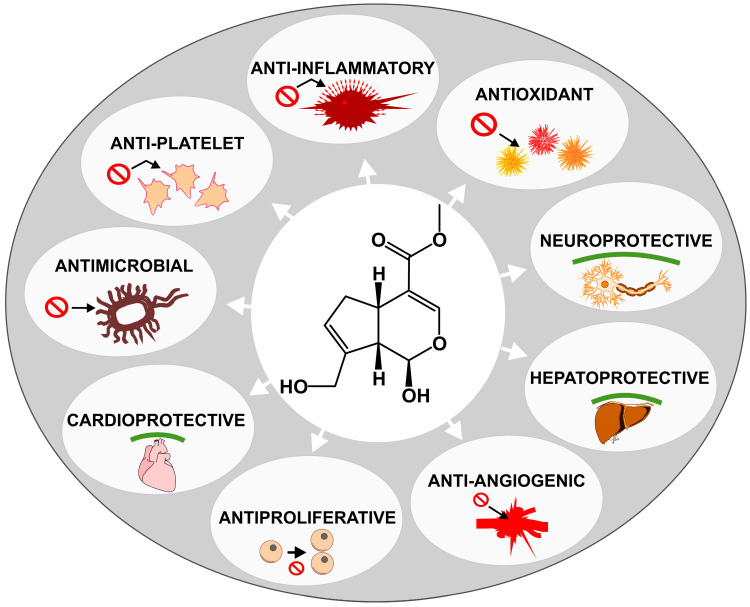
Biological activity of genipin.

The pharmacological activity of genipin (Figure 3) has been the subject of numerous in vitro and in vivo tests, in which it has been found to have anti-inflammatory, antioxidant, antimicrobial, hepatoprotective, neuroprotective, cardioprotective, antiplatelet, anti-angiogenic, and anti-proliferative effects against a variety of tumour cell lines [28].

### 3.1. Anti-Inflammatory Activity

**Figure 4:**
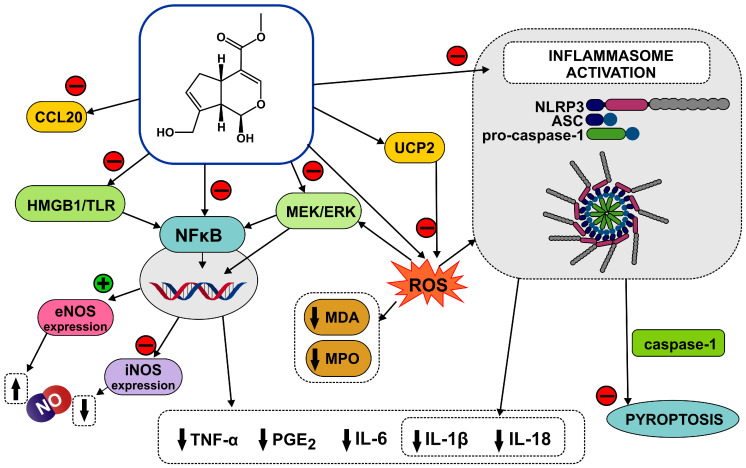
The main pathways involved in the anti-inflammatory activity of genipin. Abbreviations: ASC—apoptosis-associated speck-like protein containing a caspase recruitment domain, ERK—extracellular signal-regulated kinase; CCL20 CC—chemokine ligand 20; eNOS—endothelial nitric oxide synthase; HMGB1—high mobility group box 1 protein; IL-1β—interleukin-1β; IL-6—interleukin-6; IL-18—interleukin-18; iNOS—inducible nitric oxide synthase; MDA—malondialdehyde; MEK—dual specificity mitogen-activated protein kinase; MPO—myeloperoxidase; NFκB—nuclear factor kappa B; NLRP3—NLR family pyrin domain containing 3; NO—nitric oxide; PGE_2_—prostaglandin E; ROS—reactive oxygen species; TLR—toll-like receptor; TNF-α—tumour necrosis factor α; UCP2 uncoupling protein 2.

Genipin may prove to be an effective anti-inflammatory agent both when applied topically and with systemic effects, as found in numerous in vitro and animal model studies (Figure 4).

In in vitro studies on RAW 264.7 macrophages stimulated with lipopolysaccharide (LPS, 0.1 μg/μL), genipin applied at concentrations of 50–150 µM/mL, there was significant inhibition of NO (nitric oxide) and PGE_2_ production and reduced IL-6, IL-10 and IL-1β levels [29]. For example, the compound at a concentration of 150 µM/mL reduced IL-6 levels to 4.0 pg/mL vs. 16.3 pg/mL (control), IL-10 levels to 14.8 pg/mL vs. 84.8 pg/mL, IL-1β levels to 10.8 pg/mL vs. 43.9 pg/mL, compared to untreated control. At the same concentrations, genipin, when applied to inhibit inflammation in rotavirus-infected Caco-2 cells, also caused a significant reduction in pro-inflammatory cytokine levels and NO and PGE_2_ production [29].

In in vitro studies on RAW 264.7 macrophages stimulated with IFN-γ (10 U/mL) and LPS (1 μg/mL), genipin dose-dependently (50–300 µM/mL) inhibited NF-κB activation, iNOS (inducible nitric oxide synthase) expression and NO production [30]. It significantly reduced LPS-induced degradation of inhibitor-κB-β (I-κB-β). Similarly, in IL-1β-stimulated hepatocyte cells, it reduced iNOS levels and its mRNA expression, inhibited NO production and mRNA expression for TNF-α and IL-6 [31]. Also, in LPS-stimulated BV2 microglial cells, genipin inhibited the production of TNF-α, IL-1β, NO and PGE_2_ and the activation of NF-κB [32]. On the other hand, genipin increased endothelial nitric oxide synthase (eNOS) expression and NO secretion in endothelial cells (HUVEC), which is explained by the inhibition of exocytosis by this compound [33]. Inhibition of thrombin-induced VWF (von Willebrand factor) release and P-selectin translation in a dose- and time-dependent manner was observed. In in vivo tests, prolongation of bleeding time was observed in mice.

The anti-inflammatory activity of genipin was also tested on human periodontal ligament cells (HPDLCs) stimulated with interleukin IL-1β (10 ng/mL) [34]. Pretreatment of the cells with genipin (12.5 or 25 µg/mL) significantly inhibited the production of CCL20 (CC chemokine ligand 20) and IL-6, and caused inhibition of ERK (extracellular signal-regulated kinase) phosphorylation and the p65 subunit of NF-κB. It is hypothesised that this direction of genipin activity—inhibition of the MEK/ERK pathway in interleukin IL-1β-stimulated HPDLCs—could be exploited for the treatment of periodontal inflammation.

In in vitro tests on bone-marrow-derived macrophages (BMDMs), genipin inhibited the activation of NLRP3 and NLRC4 inflammasomes [35]. Inflammasomes are multiprotein intracellular complexes; they are composed of a sensor-like molecule (NLR or ALR family protein receptors), an adaptor protein (ASC, apoptosis-associated speck-like protein containing a caspase recruitment domain), and an effector enzyme (pro-caspase 1) [36]. They participate in infections of various origins, control of the composition of the intestinal microbiome, maintenance of intestinal mucosal homeostasis, response to cellular stress and tissue damage, among other things [37]. Inhibition of inflammasome activation leads to inhibition of the secretion of the pro-inflammatory interleukins IL-1β and IL-18, as well as inhibition of the initiation of pyroptosis (caspase-dependent lytic cell death). In BMDM cells, genipin reduced IL-1β secretion and caspase-1 activation, while it did not affect the expression of pro- IL-1β, pro-caspase-1, or ASC proteins, indicating a mechanism of action related to inhibition of inflammasome activation [35]. It is likely that, in this case, the suppression of inflammasomes may be related to uncoupling protein 2 (UCP2)-ROS signalling.

In a study of the anti-inflammatory and gastroprotective effects of genipin, an in vivo model, in which acute damage to the mouse gastric mucosa was induced by intragastric application of ethanol (15 mL/kg), has been used [38]. The test group received oral genipin at a dose of 50 or 100 mg/kg for seven days prior to injury. A significant reduction in the levels of the oxidative stress markers MDA (malondialdehyde) and MPO (myeloperoxidase) was observed compared to the untreated group: in the 50 mg/mL genipin group by 16.8% and 15.9%, respectively, and for the 100 mg/mL by 16.8% and 17.4%, respectively. Genipin inhibited ethanol-induced activation of the NLRP3/ASC/caspase-1 signalling pathway. It is suggested that genipin may be a gastroprotective agent against ethanol-induced damage by inhibiting the inflammatory response and activating the antioxidant system.

In a mouse model of endotoxaemia induced by intraperitoneal injection of LPS (10 mg/kg), intraperitoneal administration of genipin at doses of 2.5 and 5 mg/kg, immediately or 24 h after LPS administration, significantly inhibited TNF-α secretion, reduced the increase in HMGB1 (high mobility group box 1 protein), a protein involved in a number of immune and inflammatory responses, and increased mouse survival [39].

A study using a croton oil-induced acute inflammation model, showed that genipin applied at a dose of 4.42 µM/ear produced a 57.1% reduction in swelling (weight, thickness) of the mouse ear [30]. Indomethacin applied as a positive control at a concentration of 1.40 µM/ear resulted in an 83.5% reduction in oedema mass and 78.6% in thickness.

### 3.2. Antioxidant Activity

Koo et al. (2004) showed that genipin dose-dependently inhibited lipid peroxidation induced by Fe^2+^/ascorbate in rat brain homogenate (IC_50_ 936.2 µM), but lacked the ability to scavenge the DPPH radical and inhibit xanthine oxidase [30]. However, Zuo et al. (2018), using the DPPH assay, showed that genipin only possessed a slight ability to scavenge the radical [40]. The protective effect of genipin against free radical damage produced by H_2_O_2_ was tested in the ARPE-19 cell line, derived from retinal pigment epithelium (RPE) [41]. Oxidative damage to the RPE is one of the causes of age-related macular degeneration (AMD). Genipin (30 µM) reversed the suppressive effect of hydrogen peroxide (200 µM) on the Nrf2 signalling pathway. Nrf2 (nuclear factor erythroid 2-related factor 2) is a transcription factor involved in the protection against oxidative stress by, among other things, regulating reduction-oxidative processes, repairing DNA, and xenobiotic metabolism [42]. In H_2_O_2_-treated cells, genipin increased the expression of Nrf2, HO-1 (heme-oxygenase 1) and NQO1 (NAD(P)H dehydrogenase [quinone] 1). An increase in cell viability and a decrease in ROS levels and the number of apoptotic cells were observed. The authors suggested considering the use of genipin in the prevention of AMD. Genipin-mediated activation of the Nrf2/HO-1 antioxidant pathway was also observed in studies on RAW 264.7 macrophages [43].

The nephroprotective effect of genipin related to inhibition of oxidative and nitrative stress was observed in an animal model of cisplatin-induced nephropathy [44]. Cisplatin, a drug used in cancer chemotherapy, causes several adverse effects, among the most serious of which are neurotoxicity, ototoxicity and, in particular, nephrotoxicity. In tests on mice, severe tubular necrosis with dilatation, inflammation and intraluminal epithelial anoikis induced by this drug, among others, were observed. Genipin was administered to animals intraperitoneally 2 h before or 12 h after cisplatin administration. Genipin pretreatment (1, 5, 10 mg/kg) inhibited NADPH oxidase activation, increased GSH levels, increased SOD (superoxide dismutase) activity and reduced 4-HNE (4-hydroxynonenal) and 3-nitrotyrosine accumulation in the kidney. A reduction in BUN (blood urea nitrogen) and Cr (serum creatinine) levels was observed. The strongest effect was seen at a dose of 10 mg/kg. Administration of genipin (10 mg/kg) 12 h after renal injury reduced cisplatin-induced nephrotoxicity.

The neuroprotective effects of genipin against ROS (ROS-mediated injury by 1 mM tert-butyl hydroperoxide, TBHP, for 24 h, or 10 µM rotenone for 1 h) and RNS (NO-mediated stress induced by incubating OHSC in 10 mM SNAP—the direct NO donor S-nitroso-N-acetylpenicillamine, for 24 h) were tested on organotypic hippocampal slice cultures (OHSC) [45]. Genipin at a concentration of 50 µM administered at 0, 1, 2, and 6 h after rotenone-induced damage significantly reduced cell death. At the same concentration, it showed a significant protective effect against a damaging organic peroxide when treatment was delayed 1 h, 6 h, or 24 h after TBHP injury onset. Genipin also significantly reduced both SNAP-induced cell death and nitrite levels, at 24 h.

Of interest are the results of an in vitro study on the dose-dependent involvement of genipin in the antioxidant system of cancer cells [46]. For this purpose, human gastric adenocarcinoma target line (AGS) cells were treated with different concentrations of genipin (1, 12.5, 25, and 50 µM). At lower doses (up to 25 µM), genipin induced antioxidant effects via the JNK/Nrf2/ARE signalling pathway. In contrast, at higher doses (in the range 25–50 µM), it exerted cytotoxicity. The result of this study indicates that the dosage of this compound needs to be determined to achieve the desired therapeutic effect without serious side effects.

### 3.3. Cholagogic and Hepatoprotective Activity; Hepatotoxicity

The fruit of *Gardenia jasminoides*, one of the main natural sources of geniposide and genipin, has been used for centuries in Traditional Chinese Medicine for liver disorders. It is believed that genipin is one of the main components responsible for choleretic and hepatoprotective activity of this plant material [47].

It has been suggested that the choleretic effect of genipin may be related to the stimulation of the function of Mrp2 (multidrug resistance associated protein 2), a protein that mediates the secretion of glucuronic or sulphuric acid-conjugated bile acids into bile and the transport of conjugated bilirubin, organic ions and some drugs [48]. In a study in Sprague Dawley rats that received a genipin solution (1 µM/min per 100 g body weight) by intravenous infusion into a jugular vein 30 minutes after the common bile duct cannulation, a significant increase in bile flow (230%), in the secretion of biblirubin-biliary conjugates (513%) and reduced glutathione (GSH) (300–350%) was observed. However, there was no increase in bile acid secretion.

The inhibitory effect of genipin on hepatocyte apoptosis was observed in in vitro assays on primary-cultured hepatocytes isolated from BALB/c mice, by in situ perfusion to which 100 ng/mL of Jo2, a Fas receptor-activating antibody, was added [49]. Genipin preadministration also inhibited caspase 3 and 8 activation and caused a reduction in the mitochondrial transmembrane potential (ΔΨ_m_) of hepatocytes in the liver homogenates obtained from mice intravenously injected with Jo2.

In a study in C57BL/6 mice, genipin and geniposide were shown to have a protective effect against hepatic ischaemia–reperfusion (I/R) injury [50]. Animals received genipin (50 mg/kg) and geniposide (100 mg/kg) half an hour before a 60-minute liver ischaemia was induced (by clamping of the portal vein branch and hepatic artery supplying the left and middle lobes of the organ), followed by six hours of reperfusion. In I/R mice, serum alanine aminotransferase and hepatic lipid peroxidation levels were elevated, while the glutathione/glutathione disulphide ratio in the liver was reduced. I/R also caused an increase in TUNEL-positive cells, release of cytochrome c from the mitochondrial intermembrane space into the cytoplasm and activation of caspase-3. These changes were attenuated by geniposide and genipin administration. In genipin-treated I/R mice, MDA levels were approximately 0.28 nM/mg protein compared to approximately 0.41 nM/mg in untreated I/R mice. On the other hand, the GSH/GSSG ratio in genipin+I/R mice was much higher at about 6.28 compared to I/R mice at about 2.87. In genipin+I/R mice, there was also a significant increase in the expression of HO-1 which has a protective function against ischaemia–reperfusion injury through modulation of oxidative stress. The authors believe that by reducing oxidative stress, genipin, when administered orally, may play a hepatoprotective role in ischaemia–reperfusion injury, with the limitation being its poor solubility and lower stability compared to geniposide (water solubility ≥ 5 mg/mL). Geniposide, whose main metabolite, formed by intestinal bacterial activity, is genipin, is perhaps a better ’starter’ therapeutic agent.

In animal studies, genipin has been shown to be a hepatoprotective agent but, on the other hand, may have hepatotoxic effects. In studies in healthy mice and with α-naphthalene isothiocyanate-induced liver damage, genipin showed dose-dependent hepatotoxicity (125 mg/kg, 250 mg/kg, and 500 mg/kg) that increased with dose, with LD_50_ 510 mg/kg in an acute toxicity study. It is, however, important to underline that the hepatotoxicity of genipin was reversible. The authors suggest that genipin may exert its hepatotoxicity by the impairment of the UDP-glucuronosyltransferase system and cytochrome P450 enzyme activity [51].

One of the proposed mechanisms of action of genipin responsible for the hepatotoxic effect is the covalent binding of genipin to cytochrome 450 proteins—enzymes involved in the metabolism of drugs and toxins [52]. Genipin inhibited all the P450 isoforms tested. While different types of action were observed against the different forms: direct, irreversible and time-dependent (CYP2C19), irreversible and time-dependent (CYP2B6, CYP2C8, and CYP3A4 M, midazolam as substrate), mixed-type inactivation and time-dependent (CYP1A2, CYP2C9, CYP2D6, and CYP3A4 T, testosterone as substrate).

Results from network pharmacological studies indicated that the hepatotoxic effects of genipin may be related to the TNF signalling pathway, resulting in stimulation of cell apoptosis, as well as inflammation [53]. Luo et al. (2021) evaluated the toxicity of genipin to HepG2 cells, with a IC_50_ value of 207.83 ± 37.06 μM. In a subsequent metabolomic experiment, the authors suggest that this compound may induce damage to hepatocytes by impairing metabolism of certain amino acids (e.g., arginine, proline, leucine), due to its ability to form adducts. Moreover, the metabolism of pyrimidine, purine, pantothenate and CoA was also disregulated in genipin-treated cells [54]. In another study, genipin reduced the viability of rat liver BRL-3A cells (EC_50_ 154.73 ± 22.6 µg/mL), indicated cell apoptosis and necrosis, and blocked the cell cycle in the G_2_/M phase. The compound also caused an increase in aspartate aminotransferase, (AST), alanine aminotransferase (ALT), and alkaline phosphatase (ALP) levels in cells, with an observed decrease in superoxide dismutase (SOD) and glutathione (GSH) activity. In addition, genipin induced the release of pro-inflammatory cytokines, such as TNF-α, IL-6, and nitric oxide, probably as a result of binding to the pro-inflammatory factor TNFR1 receptor of the NF-κB and MAPK (mitogen activated protein kinase) signalling pathways [55].

The overall effect of genipin, together with some pro-inflammatory effect, seems to be rather limited, as the results reporting such activity are mainly based on in vitro experiments, or on a single in vivo study with relatively high doses, far exceeding the beneficial health effect observed by other authors. However, this issue should be clarified in the future, due to insufficient data available.

### 3.4. Anti-Proliferative Activity; Potential Use of Genipin in Cancer Therapy

Genipin is among the plant-derived substances investigated for its potential use in cancer therapy or its adjunctive effect on therapy with cytostatic drugs [28]. In a number of tests, mainly in vitro, it has shown antiproliferative activity against cancer cell lines, including colon, stomach and lung [56]. The results of these studies are summarised in a table (Table 1).

In most cases, induction of apoptosis was observed through effects on the mitochondrial cascade involving UCP2 (uncoupling protein 2) and STAT3 (signal transducer and activator of transcription 3) [28]. In studies on pancreatic carcinoma cells (Panc-1), a key structural element for the cytostatic effect of genipin was found to be the free hydroxyl group at C1 [56]. The substitution of hydroxyl moiety on C1 with a sugar or ethyl residue resulted in the loss of this activity.

One of the most studied mechanisms of action of genipin is its ability to specifically inhibit the UCP2 protein [79]. The UCP2 protein, located in the inner mitochondrial membrane, is responsible for uncoupling oxidative phosphorylation in mitochondria, resulting in a reduction in cellular ATP levels and ROS production. In particular, it is involved in cellular resistance to oxidative stress and regulation of free radical production by mitochondria [80]. Overexpression of UCP2 has been found in breast, ovarian, bladder, esophageal, testicular, kidney, colorectal, lung, pancreas, prostate cancers, and in leukaemia, among others [81]. Its tumour promoting properties have been demonstrated in in vitro and in vivo animal tests.

Inhibition of UCP2 in UCP2-overexpressing breast cancer cells (MCF7 UCP2) by genipin resulted in a reduction in UCP2 tumorigenic properties, reduced cell proliferation, clonogenic survival and matrigel invasion [80]. Similarly, in pancreatic (PaCa44, Panc1), lung (A-549) and prostate (DU145, PC3) cancer cells, inhibition of UCP2 function led to an increase in mitochondrial ROS production and cell growth inhibition [65,66,69].

A mechanism of Egr1/p21 regulation by genipin was proposed for gastric adenocarcinoma; human (homo sapiens) cells (AGS) [58]. Genipin induced apoptosis via the p53-independent Egr1/p21 signalling pathway in a dose-dependent manner (25–100 µM), upregulated p21 through the nuclear translocation and binding of Egr1 (early growth response 1) to the p21 promoter site. The transcription factor Egr1 is involved in modulating the expression of proteins involved in tumour initiation and progression, including growth factors, pro-inflammatory cytokines, proteinases, among others. It has been shown that its expression in a primary gastric tumour and metastases is significantly higher than in healthy cells [82].

Another mechanism of action of genipin against gastric cancer cells (including AGS and MKN45 cells) was proposed by Jo et al. [57]. Genipin-induced apoptotic cell death was shown to be associated with JAK2/STAT3-regulated Mcl-1 suppression. Activation of the JAK2/STAT3 pathway has an important function in tumour formation and transformation, for example, in lung, breast and colorectal cancers. Blocking the expression of STAT3, the phosphorylated form of which is detected in approximately 70% of cancers, results in the inhibition of tumour cell proliferation in in vitro assays and the inhibition of tumour progression in vivo [83]. In leukaemia U937 cell lines and myelogenous cell lines (U266 and U937), genipin inhibited the constitutive STAT3 activation and downregulated the expression of STAT3 target genes including Bcl-2, Bcl-xL, survivin, cyclin D1, and VEGF [74,75].

In in vitro assays genipin showed effects on intracellular signalling cascades mediated by mitogen-activated protein kinases (MAPKs), including p38 MAPK and JNK (c-Jun N-terminal kinase). These kinases are involved in regulating the activity of many transcription factors and proteins, cell division and differentiation, cell survival or apoptosis [84]. Genipin dose-dependently inhibited proliferation and induced apoptosis of human leukaemia K562 cells through activation of JNK/Fas-L signalling and G_2_/M arrest [74]. A significant increase in caspase 3 activation was observed after 6 h of treatment and a further increase in activity at 24 h. The antiproliferative effects of genipin and the reduction in AGS cell viability were also partly due to apoptosis associated with caspase 3 activation [57]. Activation of the pro-apoptotic proteins cleaved PARP (poly ADR (adenosine diphosphate ribose) polymerase), caspase 9, caspase 8, and caspase 3 was observed. In studies on the induction of apoptosis by genipin in HeLa cells, a large involvement of JNK was also noted, whose activation is associated with an increased amount of the p53 protein, and the accumulation of bax protein [73].

Kim et al. (2017) showed on a colon cancer cell line (HCT116), that genipin inhibits cell proliferation and the Sonic Hedgehog (SHH) signalling pathway [59]. The SHH protein, which plays a key role in embryogenesis, is also involved in promoting growth, metastasis and the resistance of cancer tumours to treatment with cytostatics and regulates the metabolism of cancer stem cells [85]. SHH inhibitors are one of the groups being investigated for use in cancer therapy [86].

Genipin may prove to be an effective agent for enhancing the efficacy of some anticancer drugs. By inhibiting UCP2, it exacerbated the cytotoxic activity of cisplatin against HCT116 cells [79]. Increased ROS production and consequent cell death were observed. Similarly, increased mortality of HL60/MX2 (acute promyelotic leukaemia) cancer cells was observed with the co-treatment of genipin and various doses of the cytotoxic drugs doxorubicin, epirubicin, and menadione, and the combination of genipin and elesclomol for A549 lung cancer cells [69,87]. Inhibition of STAT3 activation by genipin significantly enhanced the cytotoxicity of anticancer drugs (bortezomide, thalidomide, paclitaxel) against the U266 cell line (multiple myeloma cells) [75]. Particularly high efficacy was observed when genipin was combined with the selective reversible proteasome inhibitor bortezomide. A synergistic effect of genipin and bortezomide was observed with respect to the induction of caspase-3 activation and poly(ADP-ribose) polymerase (PARP) cleavage.

The effect of genipin on angiogenesis in the chorioallantoic membrane was also studied [30]. At a dose of 10 µg/egg, the degree of inhibition of blood vessel formation was 71.2%, which was comparable to the angiogenic effect of the positive control, retinoic acid (74.4%) applied at a dose of 1 µmol/egg. The authors recommend further research into the exploitation of the anti-angiogenic effect of genipin in combination with chemotherapy to increase treatment efficacy.

### 3.5. Antiviral Activity

Genipin was also investigated for its antiviral activity and its potential use in the prevention of rotavirus infection. Rotavirus infection is one of the most common causes of acute diarrhoea among young children. In addition to diarrhoea, the infection also causes severe vomiting, which taken together can lead to dehydration. It is estimated that up to 95% of the paediatric population under the age of 5 years has experienced a rotavirus infection at least once. In in vitro tests with human rotavirus Wa strain and simian rotavirus strain SA-11 propagated in MA-104 cells, genipin inhibited viral adsorption and penetration in pre-treatment, but inhibited assembly and release in post-treatment [29]. Additionally, a reduction in infection-induced inflammation was observed. In an in vivo study in rotavirus-infected EDIM (epizootic diarrhoea of infant mice) mice, oral administration of genipin to neonatal mice before and after virus infection reduced the duration of diarrhoea and virus excretion in the faeces and reduced intestinal epithelial damage [29].

Genipin also inhibited influenza A virus (strain A/Puerto Rico/8/1934 H1N1) replication in in vitro and in vivo assays [88]. The mechanism of the antiviral action of the compound in this case was believed to be based on activation of the AMPK-SIRT1-PGC-1 alpha signalling pathway, resulting in inhibition of MAPK (ERK, JNK, p38) and NF-κB signalling pathways. Reduced infection-induced expression of pro-inflammatory factors was also observed: IL-1 β, IL-6 and TNF-α.

It is estimated that in human cancers, oncogenic viruses are involved in approximately 12% of cases [89]. However, it is important to remember that although oncovirus infections are common, they rarely lead to the development of cancer. With the exception of EBV-associated lymphoproliferative disease, such cancers do not develop until 15–40 years after infection. The group of oncogenic viruses includes Kaposi’s sarcoma-associated herpesvirus (KSHV)—human herpesvirus-8, a member of the herpesviridae family, which is one of the main aetiological agents of Kaposi’s sarcoma and has also been implicated in primary effusion lymphoma (PEL) and multicentric Castleman’s disease (MCD) [90]. In vitro tests using the SLK-BAC16 KSHV infection system investigated the effect of genipin on KSHV latent replication, a phase in which the resulting products are likely to be involved in oncogenesis [91]. iSLK-BAC16 (KSHV-positive) cells were treated with different doses of genipin (0, 18, 36, and 72 µM). Genipin at lower concentrations promoted the KSHV latent replication by upregulating LANA (the KSHV latency-associated nuclear antigen) and also intensified the recruitment of RNA polymerase II to the KSHV latency control region. At higher concentrations (72 µM) it induced the KSHV lytic replication, resulting in a significant increase in extracellular KSHV genome copy numbers. The authors believe that genipin may be an important factor to manipulate the KSHV life cycle in KSHV latently infected cells.

### 3.6. Anti-Ageing Activity

The process of non-enzymatic glycosylation (glycation) of macromolecules such as proteins, DNA and lipids, among others, leads to their damage and entropic ageing [92]. Increased glycation is observed in, inter alia, inflammatory diseases, in hyperglycaemic conditions, in the case of reduced levels of antioxidant factors in the body, in lipid metabolism disorders, or in cigarette smokers [93]. An accumulation of AGEs (advanced glycation end products) has been implicated in the accelerated ageing process, i.e., of the skin. Genipin has been studied for its inhibition of keratin, collagen, and elastin glycation, as well as AGEs-cross-linking breaking activity [94]. In vitro tests in three types of protein–glucose glycation models showed that genipin reduced the formation of AEGs: fluorescent AGEs (F-AGEs), pentosidine, Nε-carboxymethyllysine (CML), and 3-deoxyglucosone (3DG). Inhibition ratio for the production of F-AGEs for genipin, applied at a concentration of 0.4 mg/mL, was: for elastin 96.9%, for keratin 83.6% and for collagen 71.1%; with IC_50_ in the range 0.013–0.086 mg/mL. It is hypothesised that genipin applied to the skin, e.g., in gel form, could inhibit the glycation process by blocking the carboxyl group of the intermediates and cross-linking, contributing to improved skin condition.

## 4. Metabolism and Bioavailability

**Figure 5:**
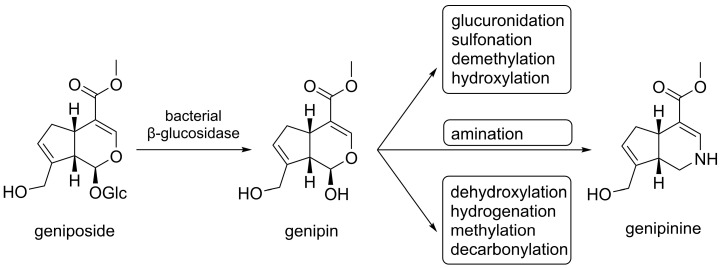
Possible main metabolic transformation pathways of geniposide.

Geniposide, present in the fruit of *Genipa americana*, when administered orally, is largely biotransformed in the gut to genipin under the influence of β-glucosidase produced by intestinal bacteria (Figure 5) [95]. Afterwards, the aglycone can be partially absorbed in the intestines and transported to the liver, where it undergoes further transformations. However, the rat liver homogenate did not hydrolyse geniposide to genipin [95]. In rat studies, its main metabolites were shown to be sulphated and glucuronidated conjugates of genipin [96].

The absorption of geniposide as a pure compound and in a traditional Chinese four-plant formulation containing *Gardenia* fruit was assessed using intestinal perfusion and Caco-2 models. In this study, geniposide had better absorption in the duodenum and jejunum, which was mainly absorbed by passive diffusion. This compound may also be a potential P-glycoprotein substrate, as verapamil affected the transport of geniposide, whereas EDTA (ethylenediaminetetraacetic acid) did not [97].

Several studies have investigated the pharmacokinetics of geniposide in rodents (rats or mice) and results suggest that the gut microflora may highly influence the bioavailability of this compound [95,97,98,99,100]. The absolute oral bioavailability of pure geniposide in rats is very low and calculated as only 9.67% [101]. On the other hand, when the *Gardenia* fruits extract was administered, the bioavailability was 32.32% [99]. The low bioavailability of geniposide might be explained its poor penetration through the gut membrane as a hydrophilic glycoside, in contrast to a lipophilic aglycone—genipin. Therefore, it has been suggested that the bioavailability of geniposide may also be low after oral administration in humans due to the presence of sulphatase in the gastrointestinal tract and liver [101].

In an in vivo pharmacokinetic study of genipin, bioavailability in rat models was shown to be high—approximately 80.2% [28]. This aglycone can be converted into various metabolites by both mammalian intestinal bacteria and microsomal enzymes of the intestinal mucosa or liver. The metabolism of genipin after oral intake leads to the formation of various glycosides and other metabolites, in particular genipin sulphate and genipin-1-O-glucuronide [96,100]. However, after intravenous bolus administration of 50 mg/kg genipin to male Sprague Dawley rats, genipin in plasma was detected mainly as genipin sulphate, with genipin 1-O-glucuronide being absent [98]. In addition, after oral ingestion of *Gardenia* fruit decoction containing geniposide, genipin sulphate was detected in the plasma, while no genipin glucuronide or genipin was detected [98]. In another study, Sprague Dawley rats were administered 250 mg/kg genipin suspended in 0.5% carboxymethylcellulose sodium solution (CMC-Na), and the metabolites of genipin present in plasma, urine, faeces and faecal fermentation were analysed by UHPC-HRMS (Ultra-High-Performance Liquid Chromatography Coupled with High Resolution Mass Spectrometry) combined with multiple data processing methods [100]. In this study, 49 metabolites were identified, formed by hydroxylation, dehydroxylation, methylation, demethylation, hydrogenation, sulphonation, glucuronidation and their overlapping reactions. The results showed that, in the process of drug metabolism, genipin usually forms intermediate metabolites that can be metabolised in many further reactions. Some metabolites of the microbiota can also be absorbed into the circulatory system [100]. Finally, studies on the human gut microbiota have shown that geniposide is also converted to the nitrogen-containing compound, genipinine [102]. After p.o. administration of geniposide to rats at a dose of 100 mg/kg, elimination was complete almost within 12 hours [101].

The distribution of geniposide in tissues varies. After oral administration of this glycoside (200 mg/kg) to rats, the highest concentration was in the kidney, followed by the spleen and liver, and the lowest in the brain. However, geniposide was determined in the brain, indicating that the glycosidic form may also cross the blood–brain barrier [101]. Penetration through the cell membrane or increased penetration of geniposide into the brain may be enhanced using other compounds that promote absorption (such as borneol), the use of complex plant extracts or technological processes (such as nanotechnology-based strategies) [28,103].

Due to genipin’s broad spectrum of activity, the bioavailability and tissue penetration of this compound is important. The metabolic reactions and their kinetics described above are crucial to better understanding the safety profile and therapeutic potential of genipin. Changes in the gut microbiota may modulate the biotransformation of geniposide in the gut, thereby altering its bioavailability [99].

## 5. Non-Therapeutic Uses of Genipin

Despite genipin’s broad spectrum of pharmacological activity, the non-therapeutic uses of this compound are of much greater interest. In some countries it is employed in the production of pigments, which are used as food additives. It is also an agent applied in dactyloscopy and tattooing. Above all, genipin is being explored as a biocompatible, biodegradable crosslinking agent in the medical and pharmaceutical industries [104].

### 5.1. Use in the Production of Blue Pigment

**Figure 6:**
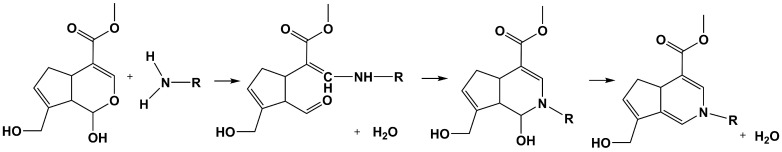
A blue pigment-forming mechanism of reaction between genipin and primary amino residue.

Today, a great many food and non-food manufacturers are trying to replace synthetic food colour additives with natural ones to meet consumer demands. Of particular interest are blue-coloured substances of plant origin, which are, however, relatively few in nature compared to, for example, yellow or red compounds. As mentioned earlier, genipin, in the presence of oxygen, reacts with primary amine groups to form a blue-coloured product. The commercially available blue pigment, gardenia (genipin) blue, is produced from genipin extracted from the fruit of *Gardenia jasminoides* [105]. The pigment is obtained by 1) microbial fermentation (using bacterial or fungal strains), hydrolysis of genipin glycosides and the spontaneous reaction of the aglycone with the amino groups, in one container, or 2) enzymatic biotransformation. In the second method, β-glucosidase added to a mixture of iridoid glycosides extracted from gardenia fruit, enables a release of genipin, which reacts with amino acids such as glycine, lysine, or phenylalanine to give a blue-coloured end-product (Figure 6) [106,107]. Although microbial fermentation is a more environmentally friendly method, the final product requires extensive purification and is considered of low value [108].

There is also a genipin-based commercial product derived from the fruit of Genipa americana—jagua (genipine-glycine) blue. In a monograph of the Joint Food and Agriculture Organization of the United Nations (FAO)/World Health Organization (WHO) Expert Committee on Food Additives (JECFA), the blue colourant jagua (genipine-glycine) blue “(…) *is obtained by reacting genipin in the filtered aqueous extract of the unripe fruit with stoichiometric amounts of glycine and heating at 70 °C for 2 h. When the reaction is complete, the product is centrifuged and concentrated and/or dried. The blue colour is due to both the polymer (average molecular weight, 6000 Da), which is composed of repeating dimers ((C_27_H_25_O_8_N_2_)_n_), and minor quantities of three dimers*” [109]. For commercial purposes, the resulting jagua (genipine-glycine) blue is additionally mixed with maltodextrin or modified starch, and no unreacted genipin is found in the final product. The blue polymer alone accounts for approximately 30–40% of jagua blue.

Gardenia blue can be found in powder form, which is mainly used for colouring fabrics, coatings or paints, and in liquid form, used as a colouring agent for food, beverages, cosmetic products and personal care products. It is widely used in some Asian countries, e.g., Japan, China, Korea, as a food additive, e.g., in jams, ice cream, noodles, flavoured fluid milk drinks, flavoured alcoholic beverages [110,111]. At the end of 2023, the U.S. Food and Drug Administration (FDA) approved jagua (genipin-glycine) blue as a colour additive in various food categories at levels consistent with good manufacturing practice (GMP).

Genipin blue pigment is more resistant to temperature and light than Indigo Blue [19]. Its colour is stable at different pH values and also at varying environmental pH values within the gastrointestinal tract, hence, for example, the observed blue colouration of the faeces of animals whose diets were supplemented with this pigment in high concentrations [112]. During storage at pH 3.6, Jagua blue showed higher stability than phycocyanin [113]. The genipin blue pigment has a characteristic absorption band with a maximum at ca. λ = ~590 nm, with minor variations depending on the type of amine [19]. In scientific publications, the pigment is assumed to have a polymeric structure (molecular weight in the range of 15–30 kDa), but due to the difficulty in isolating a single molecule, it has not yet been possible to determine the full structure and fully elucidate the course of the reaction [19,107]. Recently, studies on colourants obtained by the reaction of genipin with aniline and methylamine derivatives have suggested that the resulting products may have an open-shell configuration as determined by EPR spectroscopy [19].

The colour intensity and kinetics of the chemical reaction are strongly influenced by the type of amino acid, its concentration, and the pH of the medium [18]. The pH value directly affects the [NH^3+^/NH_2_] ratio. At acidic pH, this ratio is higher (higher NH^3+^ number), resulting in lower reactivity of the medium. A comparison of the reaction rates for lysine and arginine used to obtain the colourant at pH 6.7 showed that the reaction mixture for lysine took on a blue colour after only about 5 min, while for arginine this time was about 15 min. At this pH value, the [NH^3+^/NH_2_] ratio for arginine was about 100 times higher than for lysine. In the reaction of genipin with aromatic amines, a bathochromic effect was observed and the colour of the solution changed from blue to green [19].

In addition to the dyeing effect, some therapeutic aspects of the genipin pigment are also being considered. In an in vitro study on RAW 264.7 macrophages stimulated with lipopolysaccharide (0.2 µg/mL), the anti-inflammatory effect of different concentrations (12.5, 25, 50, 100 µM) of the blue pigment obtained by a reaction of genipin with glycine was observed [114]. The substance dose-dependently inhibited the expression of mRNA for inducible nitric oxide synthase (iNOS) and cyclooxygenase (COX-2) at the concentrations tested, reduced interleukin IL-6 production at 25, 50, 100 µM and PGE_2_ production at 50, 100 µM. TNF-α production was only slightly inhibited by blue colourant at 50 µM concentration. Reductions in plasma TNF-α and IL-6 levels were observed in in vivo studies on LPS-stimulated ICR mice that received i.p. blue pigment (30, 60, 120 mg/kg) 30 min prior to intraperitoneal administration of LPS (1 mg/kg). Its highest dose reduced TNF-α levels by 59.2% and IL-6 levels by 19.5%; in comparison, dexamethasone (10 mg/kg) caused reductions of 81.7% and 36.1%, respectively. Inhibition of signalling cascades leading to the activation of NF-kB is thought to be one of the mechanisms of action of blue pigments. The authors discuss the possibility of using this product as a food ingredient in the prevention and treatment of chronic inflammatory diseases.

The increasing interest of the food industry, in the US and Europe, in the gardenia blue pigment, raises questions about the safety of its use, especially long-term, and the concentrations that can be used in food products. The pigment obtained from the reaction of genipin and glycine in studies on RAW 264.7 cells did not significantly alter cell viability up to a concentration of 200 µM [114]. In bacterial cell reverse mutation assays, food blue pigment (gardenia blue (24.8%), dextrin (69.4%), water (4.6%), and other components (1.2%); residual genipin less than 10 ppm) tested up to 5000 µg/plate, in the presence or absence of metabolic activation, had no cytotoxic or mutagenic effect against the tested strains of *Escherichia coli* and *Salmonella typhimurium* [110]. In in vitro micronucleus and chromosomal aberration assays and in animal micronucleus and comet assays, negative results were also observed for gardenia blue. It should be added that, in bacterial cell reverse mutation assays, free genipin at ≥2000 µg/plate showed cytotoxic effects against *Salmonella typhimurium* strains, and against *Escherichia coli* at 5000 µg/plate, in the absence of a metabolic activation system [110]. No mutagenic effect was found against *Escherichia coli* WP2 uvrA pKM10 and *Salmonella typhimurium* TA98, TA100, TA1535 in the presence or absence of metabolic activation.

In a 104-week animal study, gardenia blue (obtained from the Japan Gardenia Blue Colour Industry Association) was added to the diet of rats at concentrations of 2.5 and 5% (equivalent to overall achieved intakes of 1076.9 or 2172.9 mg/kg/day for males and 1266.5 or 2533.3 mg/kg/day for females) [112]. No clinical signs and no differences in haematological parameters or mortality were observed between the test and the control group (receiving a diet without added pigment) during the experiment. Incidents of neoplastic lesions were observed in both the test and control groups with similar frequency. The animal faeces in the test group were blue in colour. These results are consistent with those of Maronpot et al. (2023) [115]. In a 24-month experiment, Sprague Dawley rats were administered 0.5%, 2.5%, or 5.0% gardenia blue (containing 32.3% gardenia blue colour, 62.7% dextrin, 3.2% water, and 1.8% other components; genipin was not detected in the powder) for 12 (chronic toxicity cohort) or 24 (carcinogenicity cohort) months. Neither toxicity nor carcinogenicity of the product was observed. In a study on the effects of orally applied gardenia blue powder (consisting of 32.3% gardenia blue colour (CAS:106441-42-3), 62.7% maltodextrin, 3.2% water, and 1.8% other) on pregnancy and embryonic and foetal development, different doses of the product (500, 1000, or 2000 mg/kg/day) were administered to female rats (from day 6 to 20 of gestation) and rabbits (from day 7 to 28 of gestation) [107]. In rats administered the product at a dose of 2000 mg/kg/day and in rabbits (at all doses), dark/blue discoloration of the kidney was observed. In contrast, blue colouration of the mesenteric lymph nodes was evident in rabbits at doses ≥ 1000 mg/kg/day, and blue colouration of the gastrointestinal tract at all doses. At the highest dose, a slight reduction in food intake was observed in both rats and rabbits. Most importantly, however, it appears that in both rats and rabbits, no adverse effects associated with the use of genipin were observed on pregnancy or foetal development. Thus, doses up to 2000 mg/kg/day were considered safe.

In addition to its use as a substrate in the production of gardenia blue, genipin is also used as a colouring substance in temporary jagua tattooing. The ‘pigment’ consists of genipin-containing juice or extract from the unripe fruit of *Genipa americana*. The dark blue-greyish or dark blue colouring on the skin appears only after genipin has bound to skin proteins [116]. These genipin-induced skin colouring properties have been known and applied by South American Indian tribes and in China for centuries. Jagua tattooing is generally considered safe; nevertheless cases of adverse reactions have been described, including a patient who developed symptoms of allergic contact dermatitis after having such a procedure several times in a short period of time (4 times in 6 weeks) [117].

Genipin has also been examined for its potential use in leather dyeing [118]. In the first stage, a chrome-free tanning process using citrate-masked aluminium sulphate (Al_2_(SO_4_)**_3_**·18 H_2_O to be the equivalent of 6.0% Al_2_O_3_ with sodium citrate ([Cit]/[Al^3+^] = 1/4) as a masking agent) was used. In the second stage—genipin tanning, aluminium pretanned samples were split to 1.0–1.2 mm. The resulting products had a dark blue, almost black colour and did not require an additional colouring step.

### 5.2. Use of Genipin as a Crosslinking Agent

Genipin was first proposed as a crosslinking agent in the work of Sung et al. (1999) [119]. It has been shown that the mechanism of genipin-mediated crosslinking can proceed via two reactions [104]. In the first, a more rapid one, there is an attack of the primary amino group, found in, e.g., amino acid, chitosan, or gelatin, on the C3 carbon of genipin, which induces the opening of the iridoid ring. This is followed by the reaction of the newly formed aldehyde group and the secondary amide with the formation of a heterocyclic ring. The second proposed mechanism is a nucleophilic substitution reaction, in which the free amino group of the polymer attacks the carbonyl group of the genipin, resulting in the formation of an amide bond. The reaction time for the genipin crosslinking reaction can be long, possibly over 70 h [120].

A comparison of genipin and the known crosslinking agent glutaraldehyde in an in vitro cytotoxicity assay (MTT assay) on 3T3 fibroblasts showed that genipin was 10,000 times less cytotoxic. In addition, it was reported that the proliferative ability of cells after exposure to genipin was approximately 5000 times higher than after exposure to glutaraldehyde [119]. It has been suggested that genipin, as a compound of very low toxicity, could replace glyoxal (mutagenic properties) or glutaraldehyde (neurotoxic) used in materials engineering [121]. Although the vast majority of studies prove the safety of genipin, there are also reports of cytotoxic effects caused by too high a concentration of this iridoid [122,123].

Genipin is a valuable agent for 3D crosslinking, thereby increasing the structural integrity and stability of biopolymers, e.g., chitosan and gelatine, which translates into increased mechanical strength, and can also reduce shrinkage (e.g., collagen) [104]. Examples of the use of genipin as a crosslinking agent are summarised in a table (Table 2).

Genipin is of particular interest to manufacturers of dressings, drug manufacturers (drug carrier system component) and food packaging manufacturers, as well as in tissue engineering. The crosslinking of biopolymers using genipin produces blue-coloured hydrogels, films, sponges, and scaffolds. Different concentrations of genipin used in the crosslinking process affect the mechanical properties and swelling capacity of the hydrogels [120]. In the case of a chitosan hydrogel, the use of 0.5% (*w*/*v*) genipin led to the cross-linking of approximately 33% of the free chitosan groups, while at a concentration of 1% (*w*/*v*) genipin, the hydrogel was 60% cross-linked [124]. Increasing the genipin concentration from 0.5% to 2% increased the compression resistance of the hydrogel [120]. At the same time, an increase in pore size from 180 to 1100 μm^3^ was observed. Concentrations above 2% resulted in a decrease in the mechanical properties of the hydrogel and the formation of pores with irregular walls.

The hydrogel cross-linked with 2% genipin had adequate mechanical properties for use in articular cartilage and intervertebral disc repair. It is a result of a significant increase in the strength (compressive strength) of the modified hydrogel, which normally presents weak mechanical strength, due to high cross-linking ability of genipin [120]. The network of links is optimised to mimic the tissue it substitutes. Additionally, it promoted chondrogenic differentiation of the grafted cells. In the case of genipin-cross-linked chitosan/collagen, optimal mechanical, structural, and biological properties were obtained using 1.0% genipin, and the recommended temperature for the cross-linking process was 20 °C [125].

Cross-linking with genipin increases the stability and activity of substances used in bone regenerative medicine, such as collagen. This phenomenon was observed in studies on Wharton’s Jelly, a mucoid, jelly-like connective tissue found in the umbilical cord, in which collagen is one of the basic components [126]. In addition to collagen, this substance contains mesenchymal stromal cells in an extracellular matrix in which growth factors and polymers such as hyaluronic acid are present. Despite the promising biological effects, the use of Wharton’s Jelly in regenerative therapy poses difficulties due to complete resorption of the components within three weeks of administration, as well as unsatisfactory elasticity. Cross-linking the collagen fibres with genipin increased the durability of the biopolymer by increasing its resistance to collagenase. The authors of the cited study noted the potential for adverse effects when using higher doses of genipin.

Genipin is particularly popular as a crosslinking agent via covalent crosslinking in the chemical modification of chitosan. The resulting three-dimensional polymer network can be used in the manufacture of dressings for hard-to-heal wounds or for the construction of bioscaffolds for the reconstruction of human tissues and organs [127]. The properties of genipin-crosslinked chitosan nanofilms are highly pH-dependent [128]. At pH 3, swelling of the nanofilm was observed, while shrinkage was observed at pH 6. An increase in elastic modulus was also observed from ≈500 MPa at pH 3 to ≈700 MPa at pH 6.

Chitosan cross-linked with genipin was used, inter alia, to obtain a polymeric cell–substrate in the form of a membrane for the culture of corneal ocular epithelial cells [129]. In order to improve the mechanical properties of the membrane, keratin was introduced into the chitosan–genipin system. The same team also prepared a fully biodegradable culture medium consisting of a genipin-cross-linked chitosan-based system, which was enriched with collagen [130]. The resulting material is intended to be a readily available and cheap substitute for an amniotic membrane. On the anterior chamber of a rabbit eye model, it was shown that implants made of genipin-crosslinked chitosan did not induce inflammation, whereas glutaraldehyde-crosslinked chitosan up-regulated IL-6 gene levels [121].

Another direction of R&D into the application of genipin-crosslinked biopolymers is their use as substitutes for non-degradable plastics such as polyesters, polyamides, or polyethylenes. This applies in particular to genipin-crosslinked chitosan, gelatine, or soya proteins (Table 2). Such ‘bio-plastics’, in addition to the favourable physico-chemical properties characteristic of plastics, do not pose a threat to the environment due to their biodegradability. The structural modification obtained by the addition of genipin is responsible for the mechanical strength and maintenance of the material’s integrity. Furthermore, this type of material keeps moisture levels low enough, which translates into the durability of products stored in packages made from it.

Incorporating antioxidant (e.g., astaxanthin) or antimicrobial (e.g., thymol) substances into the structure of such materials additionally has a positive effect on extending the shelf life of stored products (Table 2) [131,132]. The lack of toxicity of genipin at the concentrations used for crosslinking makes it possible to obtain edible packaging with the use of polymers that are also food products, such as gelatine [104]. Another important activity from the point of view of recycling and resource reuse policy is, for example, the recovery of keratin from animal waste from slaughterhouses (feathers, scraps). The structure of keratin-polyvinyl alcohol-tris(hydroxymethyl)aminomethane films, enhanced by the addition of the cross-linker genipin, is suitable for use in the production of packaging [133].

Similarly, genipin-based drug delivery systems are promising alternatives in the pharmaceutical technology. Manufacturers are interested in the involvement of genipin in diverse forms of drugs application, such as buccal patches, hydrogels, nanoemulsions, nanoparticles, and more complex systems. It is mainly due to high biocompatibility, safety, and the availability of genipin. Furthermore, the development of the desirable material is achievable and the characteristics of the obtained forms (e.g., controlling of the drug release) are satisfactory (see Table 2 for examples).

**Table 2:** Examples of the use of genipin as a crosslinking agent.

Basic Material	Genipin (GN) Cross-Linked Material	Effect of Genipin (GN) Cross-Linking	Application	Ref.
Chitosan				
	Chitosan blended with polyvinyl alcohol to produce scaffolds	- The degradation rate was retarded: from 3 to 7 days	Liver tissue regeneration	[134]
				
	Curcumin-loaded chitosan-polyvinyl alcohol films	- Mechanical attributes improved: tensile strength of films 18.53 MPa (0% GN) to 27.99 MPa (0.5% GN) - Structural integrity kept even after the swelling with appropriate moisture amidst the wound retained - The time of wound healing decreased (in rats)	Wound dressing	[135]
				
	Chitosan linked to physically cross-linked peptide with indomethacin	- The strength of the hydrogels significantly meliorated: storage modulus from 69 Pa to 4630 Pa - The degradation rate (from 67% to 18% during 24 h) of the peptide and releasing the indomethacin was retarded (prolonged lifetime of the wound dressing and extended release of the Arg-Gly-Asp tripeptide and indomethacin) - Wound closure after 15 days	Wound dressing	[136]
				
	Diclofenac sodium loaded chitosan hydrogels	- Enterically protected hydrogels obtained (the release of the diclofenac took place in the intestine)	Drug delivery vehicles	[137]
				
	Chitosan constituting one of the four electrospun layers included in the ibuprofen and antibiotic-loaded complex buccal patches	- The mechanical attributes of the buccal patches increased - The swelling (critical for mucoadhesion) of the patches was modulated: the muco-adhesion strength was increased from 21.37 to 39.11 g - The release of ibuprofen was retarded	Drug delivery vehicles	[138]
				
	Chitosan–glycerol–phosphate disodium salt thermo-responsive hydrogel, deposited onto silk	- The pores of the hydrogels were narrowed from 50 μm to 5 μm, contributing to the effect of controlled, prolonged release of the paracetamol from the transdermal patches - The mechanical attributes of the hydrogels improved: compressive strength incremented from ≈1 kPa to 10 kPa	Drug delivery vehicles	[139]
				
	Thymol-incorporated chitosan-polyethylene oxide nanofibers	- The mechanical attributes (tensile strength and elastic modulus) improved - The films were rigidified, and their mobility was lowered	Packaging supplies	[132]
				
	Astaxanthin-incorporated chitosan film	- The tensile strength was improved: 10.17 MPa (0% GN) till 17.82 MPa (1.5% GN) - The water transmission rate was decreased: 4.83 g/h m^2^ (×10^–6^) (0% GN) to 4.55 g/h m^2^ (×10^–6^) (0.5% GN) and then to 4.47 g/h m^2^ (×10^–6^) (1% GN), but in the case of 1.5%—increased	Packaging supplies	[131]
				
	β-galactosidase-loaded porous chitosan beads	- The mechanic stability increased	Enzyme immobilisers	[140]
	Theaflavin-3,3′-digallate loaded chitosan-casein phosphopeptides nanoparticles	- The pH stability was increased with consequent size stability in acidic environment - The epithelial permeation of theaflavin-3,3′-digallate was increased (chitosan activity)	Drug delivery vehicles	[141]
				
Chitosan and gelatin				
	Curcumin-loaded chito-oligosaccharides/poly-ɣ-glutamic acid nanoparticles impregnated within GN cross-linked gelatin films	- The stability of nanoparticles was increased - The release of the curcumin was retarded	Drug delivery vehicles	[142]
				
	Rosemary essential oil and quercetin-incorporated chitosan-gelatin films	- The tensile strength was improved from 77.3 MPa to 86.7 MPa	Packaging supplies	[143]
				
Chitosan and collagen				
	Chitosan-collagen hydrogel impregnated with cefotaxime sodium loaded silver nanoparticles	- >98% of wounds closed after 2 weeks (in rats)	Wound dressing	[144]
				
Cellulose				
	Acetylcholinesterase-immobilised aminated cellulose fibres	- Outstanding operational stability obtained	Enzyme immobilisers	[145]
				
Cellulose and gelatin				
	Gelatin-aminografted microfibrillated cellulose hydrogels	- The mechanical attributes were improved - The structural integrity kept even after the swelling	Wound dressing	[146]
				
Hyaluronic acid				
	Hyaluronic acid-based cryogels	- Lamellar porous construction with pores size proper for cell culture was obtained	Tissue engineering Drug delivery vehicles Wound healing	[147]
				
Alginate				
	Alginate beads	- Biogenic amines (responsible for food spoilage) presence was detected by blue colour of the pigment produced in the reaction between GN and amine residues od the biogenic amines	Food spoilage sensors	[148]
				
Collagen				
	Collagen gel impregnated with gingival fibroblasts and seeded on top, with gingival keratinocytes	- The contraction was significantly lowered	Tissue regeneration	[122]
				
	Collagen in Wharton’s Jelly films	- The degradation rate (via collagenase and hyaluronidase) was retarded - The mechanical attributes were improved	Tissue regeneration	[126]
				
	Porcine small-intestinal submucosa (de-cellularized extracellular-matrix containing 90% collagen)	- The degradation rate via collagenase was retarded (from 7.48% to 3.50%) - The mechanical attributes were improved (scaffolds tensile strength from 6.26 MPa to 9.95 MPa)	Tissue regeneration	[149]
				
Chondroitin sulphate, Collagen, fibronectin, and laminin				
	De-cellularized nerve extracellular matrix-CHS (NECM-CHS) scaffolds	- The degradation rate was retarded: dissolution after 14 days decreased from 56.12% to 27.36% (vs 30.46% for glutaraldehyde) - The porosity was increased from 74.48% to 89.07%	Tissue regeneration (nerve regeneration)	[150]
				
Gelatin				
	Freeze-dried scaffolds constituted from a uniform gelatin-polycaprolactone nanofibers dispersion	- The degradation rate was retarded - The porosity of the material insignificantly reduced from 98.8% (0% GN) to 98.2% (2.5% GN) - Mechanical attributes were improved: Young’s modulus from 6.5 kPa (0.5% GN) to 6.7 kPa (1.0% GN), and 7.8 (2.5% GN)	Tissue regeneration (kidneys, cardiac muscles, intestines)	[151]
				
	Gelatin films impregnated with cinnamon oil-loaded Pickering emulsion	- The mechanical attributes were improved: tensile strength increased from 3.54 MPa (0% GN) to 20.22 MPa (5% GN), elongation percent also increased - The barrier attributes were improved: water vapour permeability was lowered from 0.48 × 10^–8^ g cm^−1^ s^−1^ Pa^−1^ to 0.24 × 10^–8^ g cm^−1^ s^−1^ Pa^−1^	Packaging supplies	[152]
				
	Residual gelatin remained after the fabrication of the soft capsules	- The mechanical attributes were improved: tensile strength increased from 2.72 MPa to 4.23 MPa - The barrier attributes were improved: water vapour permeability was lowered from 1.02 to 0.88 g mm/h^−1^ m^2^ kPa	Packaging supplies	[153]
				
	β-galactosidase-immobilised gelatin—sodium alginate beads	- The operational and storage stability was obtained	Enzyme immobilisers	[154]
				
Other peptides				
	Curcumin nano-encapsulated into human serum albumin grafted with tannic acid	- The size of the nanoparticles was stable - The encapsulation of curcumin was more efficient - The release of curcumin was better regulated - The treatment efficacy was better (in mice)	Drug delivery vehicles	[155]
				
	Casein phosphopeptides nanoparticles	- The stability of nanoparticles was increased in gastric acidic and enzymatic environment, but slightly less in intestinal fluid - The negatively charged nanoparticles aggregated preferentially at the site of inflammation in colon (in mice)	Drug delivery vehicles	[156]
				
	Neratinib and silibinin-loaded bovine serum albumin nanoparticles	- The efficacy of cancer treatment was meliorated (in mice)	Drug delivery vehicles	[157]
				
	Mussel adhesive proteins (MAP) impregnated with magnetic nanoparticles (iron oxide) microparticles	- The microparticles successfully localised in the oesophagus by an external magnetic field	Drug delivery vehicles	[158]
				
	Soy protein isolate-glycerol films	- The mechanical and barrier attributes were improved: tensile strength increased from 3.22 MPa (0% GN) to 3.28 MPa (0.1% G) and then to 4.16 MPa (1% GN), percent of elongation: from 22.53% (0% GN) to 26.71% (0.1% GN) and then to 45.84% (1% GN); further increasing concentration did not result in significant improvement - The barrier attributes were improved: the water vapour permeability was lowered from 2.41 to 1.88 × 10^−10^ g m Pa^−1^ s^−1^ m^−1^	Packaging supplies	[159]

GN—genipin.

### 5.3. Genipin in Physicochemical Analysis

Genipin has also proved to be useful for visualising fresh fingerprints on absorbent surfaces (e.g., paper) with good contrast and resolution [160]. In white light, the traces take on a blue–violet colour, and intense fluorescence can also be observed at 590 nm using a 620 nm cut-off filter; one study used a wavelength of 555 nm and a 630 nm cut-off filter [160,161,162]. For the analysis, a solution of genipin in HFE 7100 was used, in which the test surfaces were immersed, and after evaporation of the solvent, the samples were placed in an air-conditioned chamber (temperature about 80 °C and relative humidity about 65% RH) [160]. In later studies, the vacuum technique of genipin sublimation was used (1 Pa, 140 °C; followed by exposure to heat and humidity, temp. 80 °C, 80% RH for 1 h), which has the advantage of short sensitisation time, has a relatively low cost and helps to avoid loss or degradation of samples for genetic studies [163,164].

The ability of genipin to link to amine residues with the simultaneous formation of a blue colour is the basis for the use of this iridoid for qualitative–quantitative colourimetric analyses, e.g., of amino acid mixtures, as well as for distinguishing chitin from chitosan by calculating the amount of D-glucosamine de-coupled from the chitin chain [165,166]. For amino acids, it can provide an alternative to the commonly used ninhydrin reaction. The main differences between the two reagents are that genipin initiated more sensitive colorimetric detection of amino acids than ninhydrin. Furthermore, it produced more stable complexes with amino acids than ninhydrin did complexes with amino acids (the TLC spots lasted even for months) and genipin-induced reaction was almost unaffected by the presence of Cu^2+^ and Fe^3+^ ions in contrast to ninhydrin reaction (absorbances were decreased by 50 and 98%, respectively). Furthermore, it has been shown that genipin can be used to assess organophosphorus pesticides (e.g., chlorpyrifos) [145]. Initially, its role was to activate amino cellulose fibres in order to bind with acetylcholinesterase, which is blocked by this type of pesticide. However, it turned out that the combination of genipin with modified cellulose led to the formation of a fluorophore, and fluorescence was observed in addition to the formation of the characteristic blue colourant. The addition of acetylthiocholine iodide, as the substrate for the enzyme, resulted in the formation of thiocholine, in turn quenching the fluorescence by binding to the nitrogen centres. When blocked in the presence of chlorpyrifos, the enzyme did not lead to the formation of thiocholine, so the concentration of the pesticide was directly proportional to the fluorescence obtained.

## 6. Summary

Genipin is a small-molecule non-glycosidic iridoid with a broad spectrum of biological activity and with great potential for therapeutic application and beyond. In recent years, research into the pharmacological activity of genipin has focused on the mechanisms of its anti-inflammatory, hepatoprotective, antiproliferative, and antioxidant effects. While many studies have demonstrated a significant biological effect, on the other hand, there have been reports of possible hepatotoxicity. Therefore, an extremely important problem to be solved remains the determination of doses of this compound, which could be effectively and at the same time safely used in the prevention and therapy of various diseases, especially those of inflammatory origin.

In addition, studies on the bioavailability of genipin and the possibility of drug–drug interactions should be extended due to, among other things, genipin’s binding to cytochrome 450 proteins.

When discussing the advantages of natural crosslinking agents such as genipin, their benefits in relation to both the patient (biocompatibility, low cytotoxicity, often additional therapeutic effect) and the environment (biodegradable, environmentally safe) are often emphasised [167]. On the other hand, the isolation of this compound from plant sources generates high costs. Nevertheless, genipin is believed to be a valuable agent for 3D networking thus increasing the structural integrity and stability of biopolymers [104]. It is of particular interest to dressing manufacturers, tissue engineering, drug manufacturers (drug carrier system component) and food packaging manufacturers.

One of the most rapidly growing, genipin-related sectors is the Gardenia Blue market. Due to increasing consumer demand and requirements, technological advancements and regulatory changes governing the use of this pigment, opportunities for the use of the blue genipin pigment in various food, textile, or pharmaceutical industries are increasing. The Gardenia Blue market is estimated to grow from $9.68 billion in 2024 to $20.08 billion by 2031 [168].

In addition to its great potential for use as a crosslinking agent, as a substrate in pigment production or because of its therapeutic effect, genipin also has great advantages as a means of visualising various compounds due to its dual effect of colour and fluorescence.

## Figures and Tables

**Table 1:** Mode of action of genipin anti-proliferative activity against cancer cell lines.

Cancer Type by Site of Origin	Cancer Cell Line	Mode of Action	Ref.
		in vitro	
gastric cancer	AGS MKN45 MKN74 SNU-638	apoptosis induction via suppression of STAT3/JAK2/Mcl-1 pathway genipin-induced increase in cleaved PARP, caspase 3, caspase 8, and caspase 9 levels	[57]
		in vitro	
	AGS	apoptosis induction via p53-independent Egr1/p21-mediated apoptotic pathway inhibition of cyclin-dependent kinases	[58]
		in vitro	
colorectal cancer	HCT116 SNU-283	inhibition of the Hedgehog signaling pathway apoptosis induction via activation of p53/NOXA signaling	[59]
		in vivo	
	HCT116	apoptosis induction in a human xenograft model (HCT116 cells were implanted subcutaneously into BALB/c nude mice)	[59]
		in vitro	
	HCT116	apoptosis and cell cycle arrest in the G_0_/G_1_phase induction p53/Bax-mediated signaling pathway induction increase in ROS generation	[60]
		in vivo	
	HCT116 SW480	inhibition of tumor growth	[60]
		in vitro	
	HCT116	suppression of HIF-1α accumulation under hypoxia suppression of VEGF expression and the invasion of colon cancer cells via blocking the extracellular signal-regulated kinase signaling pathway	[61]
liver cancer		in vitro	
	Hep3B FaO	apoptosis induction via NADPH oxidase-dependent generation of ROS and JNK activation	[62]
		in vitro	
	FXT-induced HepG2	cancer cells migratory distance reduction inhibition of the FXT-induced cell metastasis and invasion	[63]
pancreatic cancer		in vitro	
	PaCa44	UCP2 inhibition ROS-mediated cell growth inhibition	[64]
		in vitro	
	PaCa44 Panc1	UCP2 inhibition increase in mitochondrial superoxide production	[65]
prostate cancer		in vitro	
	DU145 PC3	UCP2 inhibition decrease in intracellular pyruvic acid content decrease in succinate dehydrogenase (SDH) activity	[66]
bladder cancer		in vitro	
	T24 5637	genipin-induced cell cycle arrest in the G_0_/G_1_phase promotion of apoptosis via mitochondrial pathway decrease in phosphorylation levels of PI3K and AKT	[67]
lung cancer		in vitro	
	H1299	genipin-induced apoptosis by an increase in phosphorylated p38 MAPK expression increase in Bax expression and cytochrome c release	[68]
		in vitro	
	A-549	UCP2 inhibition glucose uptake reduction stimulation of mitochondrial ROS production	[69]
breast cancer		in vitro	
	MDA-MB-231	apoptosis induction by down-regulation of Bcl-2, up-regulation of Bax and proteolytic activation of caspase-3 activation of p38 MAPK and JNK, but not ERKs inhibition of cell growth and the invasive/migratory phenotypes	[70]
		in vitro	
	MCF-7 T47D	UCP2 inhibition increase in mitochondrial ROS production	[71]
		in vitro	
	T47D	decrease in 18F-FDG uptake, glycolytic flux and mitochondrial oxidative respiration	[72]
		in vitro	
cervical cancer	HeLa	apoptosis induction and cell arrest at G_1_ phase activation of c-Jun NH_2_-terminal kinase and p53protein	[73]
leukemia		in vitro	
	P-388 K562	apoptosis induction and cell arrest at G_2_/M phase activation of JNK pathway upregulation of Fas ligand expression	[74]
		in vitro	
	U937	suppression of constitutive STAT3 activation	[75]
multiple myeloma		in vitro	
	U266	suppression of constitutive STAT3 activation via repressing the activation of c-Src, but not JAK1 decrease in phosphorylation of STAT3 inhibition of STAT3 - DNA binding at 50 or 100 μM, but not at 25 μM	[75]
oral squamous cell carcinoma (tongue squamous carcinoma)		in vitro	
	SCC-25 SCC-9	downregulation of phosphorylation levels of PI3K, AKT, and mTOR increase in protein levels of cleaved-caspase-3 and cleaved-PARP apoptosis induction and autophagy promotion	[76]
		in vivo	
		tumor size and weight reduction	[76]
		in vitro	
	HN22 HSC-4	reduction of p-STAT3^Tyr705^ expression and its downstream target molecules, survivin and Mcl-1 induction of apoptotic cell death	[77]
glioblastoma		in vitro	
	U87MG A172	increase in mitochondrial ROS levels apoptosis induction through caspase-3 upregulation	[78]

Abbreviations: AKT—protein kinase B, ERKs—extracellular signal-regulated kinases, 18F-FDG—fluorodeoxyglucose F18, FXT—fluoxetine, JAK1—Janus kinase 1, JAK2—Janus kinase 2, JNK—c-Jun N-terminal kinase, MAPK—mitogen activated protein kinase, Mcl-1—induced myeloid leukaemia cell differentiation protein, NOXA—pro-apoptotic member of the Bcl-2 protein family, PARP—poly ADR (adenosine diphosphate ribose) polymerase, PI3K—phosphoinositide-3-kinase, STAT3—signal transducer and activator of-transcription 3, VEGF—vascular endothelial growth factor.

## Abbreviations

18F-FDG	fluorodeoxyglucose F18
3DG	3-deoxyglucosone
4-HNE	4-hydroxynonenal
AGEs	advanced glycation end products
AGS	gastric cancer cells
AKT	protein kinase B
ALP	alkaline phosphatase
ALT	alanine aminotransferase
AMD	age-related macular degeneration
ARE	antioxidant response element
ASC	apoptosis-associated speck-like protein containing a caspase recruitment domain
AST	aspartate aminotransferase
Bcl-2	anti-apoptotic member of the Bcl-2 protein family
Bcl-xL	B-cell lymphoma-extra large protein
BMDM	bone-marrow-derived macrophages
BUN	blood urea nitrogen
CAS	Chemical Abstract Services
CCL20	CC chemokine ligand 20
CMC-Na	carboxymethylcellulose sodium solution
CML	N^ε^-carboxymethyllysine
CoA	coenzyme A
Cr	serum creatinine
DMSO	dimethyl sulfoxide
DPPH	2,2-diphenyl-1-picrylhydrazyl radical
EBV	Epstein–Barr virus
EC_50_	half maximal effective concentration
EDIM	epizootic diarrhoea of infant mice
EDTA	ethylenediaminetetraacetic acid
EHSE	enzymatic hydrolysis and simultaneous extraction
eNOS	endothelial nitric oxide synthase
EPR	electron paramagnetic resonance (spectroscopy)
ERK	extracellular signal-regulated kinase
F-AGEs	fluorescent advanced glycation end products
FAO	Joint Food and Agriculture Organization of the United Nations
Fas-L	Fas ligand
FDA	Food and Drug Administration
FXT	fluoxetine
GMP	good manufacturing practice
GSH	glutathione
GSSG	glutathione oxidised
HFE 7100	methoxyperfluorobutane
HMGB1	high mobility group box 1 protein
HO-1	heme-oxygenase 1
HPDLCs	human periodontal ligament cells
HUVEC	human umbilical vein endothelial cells
I/R	hepatic ischaemia–reperfusion
IC_50_	half maximal inhibitory concentration
ILEIH	ionic liquid-based enzyme-assisted extraction coupled with in situ hydrolysis
iNOS	inducible nitric oxide synthase
IUPAC	International Union of Pure and Applied Chemistry
I-κB-β	inhibitor-κB-β
JAK1	Janus kinase 1
JAK2	Janus kinase 2
JECFA	Joint FAO/WHO Expert Committee on Food Additives
JNK	c-Jun N-terminal kinase
Jo2	Fas receptor-activating antibody
KSHV	Kaposi’s sarcoma-associated herpesvirus
LANA	KSHV latency-associated nuclear antigen
LD_50_	median lethal dose
LPS	lipopolysaccharide
MAP	mussel-adhesive-protein
MAPK	mitogen activated protein kinase
MCD	multicentric Castleman’s disease
Mcl-1	induced myeloid leukaemia cell differentiation protein
MDA	malondialdehyde
MEK	dual specificity mitogen-activated protein kinase
MPO	myeloperoxidase
mRNA	messenger RNA
Mrp2	multidrug resistance associated protein 2
MTT assay	(3-(4, 5-dimethylthiazolyl-2)-2, 5-diphenyltetrazolium bromide) assay
NADPH	nicotinamide adenine dinucleotide phosphate, reduced form
NECM-CHS	de-cellularized nerve extracellular matrix-CHS
NF-κB	nuclear factor kappa B
NLRC4	NLR family CARD domain-containing protein 4
NLRP3	NLR family pyrin domain containing 3
NO	nitric oxide
NOXA	pro-apoptotic member of the Bcl-2 protein family
NQO1	NAD(P)H dehydrogenase [quinone] 1
Nrf2	nuclear factor erythroid 2-related factor 2
OHSC	organotypic hippocampal slice cultures
P450	cytochrome P450
PARP	poly ADR (adenosine diphosphate ribose) polymerase
PEL	primary effusion lymphoma
PI3K	phosphoinositide-3-kinase
R&D	research and development
RAW 264.7	a macrophage-like, Abelson leukaemia virus-transformed cell line derived from BALB/c mice
RH	relative humidity
RNS	reactive nitrogen species
RPE	retinal pigment epithelium
SHH	Sonic Hedgehog
SLK	Kaposi’s sarcoma-derived *cell* line
SNAP	S-nitroso-N-acetylpenicillamine
SOD	superoxide dismutase
STAT3	signal transducer and activator of transcription 3
TBHP	tert-butyl hydroperoxide
TLR	toll-like receptor
TNFR1	tumour necrosis factor receptor 1
TNF-α	tumour necrosis factor α
TUNEL	terminal deoxynucleotidyl transferase dUTP nick end labelling
UCP2	uncoupling protein 2
UHPC-HRMS	ultra-high-performance liquid chromatography coupled with high resolution mass spectrometry
VEGF	vascular endothelial growth factor
VWF	von Willebrand factor
